# A systematic review on career interventions for high school students

**DOI:** 10.3389/fpsyg.2024.1461503

**Published:** 2024-12-02

**Authors:** Danqi Wang, Yanling Li, Gang Wang

**Affiliations:** School of Education, Huainan Normal University, Huainan, China

**Keywords:** career intervention, career counseling, high school students, systematic literature review, theoretical framework

## Abstract

**Introduction:**

This study presents a systematic literature review of career interventions for high school students, exploring the theoretical framework, intervention modality, evaluation and outcomes of the intervention.

**Methods:**

Five keywords, three databases and five inclusion criteria were defined. Of the 419 documents collected, 25 remained for meta-synthesis.

**Results:**

The results showed that (1) the theoretical framework mainly consisted of career construct theory, social cognitive career theory, and cognitive information processing theory. (2) The intervention modality was mainly group counseling (8 sessions, 45 min). (3) The evaluation system mainly consisted of a pre-and post-test for the experimental and control group, and (4) the intervention outcomes were mainly focused on the positive development of career adaptability and career decision-making.

**Discussion:**

The discussion offers recommendations for future research and practice in high school career interventions. (1) Social cognitive career theory should be given attention. (2) A three-tiered high school career development guidance model of career curriculum, career group counseling and individual counseling could be developed. (3) Career interventions should be shifted to mixed quantitative and qualitative studies, tracer studies, and special groups. (4) Outcome expectations should be given equal attention.

## 1 Introduction

The rapid changes of the times have posed great challenges in the career field. Brittleness, Anxiety, Nonlinearity and Incomprehensibility have become the typical characteristics of the current career era, which is also known as the “BANI” (initials) era. The BANI era emphasizes a more complex and unpredictable context, implying the need to deal not only with changes in the external environment, but also with internal anxieties that require more precise understanding and response (Godoy and Filho, 2021). On the other hand, borderless is also a key feature of today’s career development, and mobility is crucial to the borderless career era. A borderless career is one in which the career path may involve a range of employment opportunities that transcend the boundaries of a single employment setting. The main feature of “borderless” is the mobility and traversability of organisations, where an individual’s career is no longer fixed. In contrast to the stability and certainty emphasized by traditional vertically upwardly mobile careers, borderless careers emphasize horizontal mobility and boundary crossing (Defilippi and Arthur, 1996). The complexity of the borderless era is an urgent need for high school students to enhance their career education. Therefore, we need to shift to a new theoretical perspective to view and guide high school career education in this era.

Career interventions have changed from career guidance and career education to career counseling. In the 19th century, career guidance was centered on Parsons’ “person-job matching” model, where rational decision-making was based on information about oneself and career. After entering the 20th century, career education is based on career development theory, focusing on how individuals make decisions, a process-oriented career intervention. Moreover, beginning in the 21st century, career intervention is centered on career construct theory, focusing on personality traits, career adaptability and life themes, emphasizing constructing careers. These three career interventions have unique value depending on the different needs: career guidance to determine person-job fit, career education to promote career development, and career counseling to design life (Savickas, 2011).

Choi et al. (2012) used meta-analysis to explore the relationship between career decision-making self-efficacy and related variables based on Social Cognitive Career Theory (Choi et al., 2012). The theory model showed that career decision-making self-efficacy was significantly related to self-esteem, career identity, peer support, outcome expectations, and career indecision. The meta-analysis confirmed the effectiveness of career interventions in improving career adaptability and career decision-making self-efficacy. Specifically, effect sizes were higher for career decision-making self-efficacy (Whiston et al., 2017). Two recent systematic reviews have considered the broader relationship between career and other variables. Vashisht et al. (2021) argued that students with emotionally controlled and certain personality traits were more capable of improving their career adaptability based on the systematic review results (Vashisht et al., 2021). Stead et al. (2022) examined career adaptability and career decision-making self-efficacy, suggesting the need to consider participants’ country, average age, and career adaptability measurement tools in social cognitive career theory and career construction theory (Stead et al., 2022).

Early meta-analyses of career interventions focused on which were more effective, but no consistent conclusions have been reached (Oliver and Spokane, 1988; Whiston et al., 1998). Whiston et al.’s (2003) meta-analysis compares different modes of intervention. There were minimal differences between individual career counseling, group counseling and career courses, but computer-assisted intervention was less effective than the other modalities. The three critical elements of the meta-analysis were counselor support, values clarification, and psycho-educational interventions (Whiston et al., 2017). A meta-analysis of undergraduate and graduate student populations indicated that constructivist theories were more effective interventions than individual-environmental matching theories (Langher et al., 2018).

Therefore, career interventions of high school students should be focused on, and expanding the population of studies for career intervention meta-analyses is necessary. A retrospective study was conducted, and this review will include articles published from 2014 to 2024. A synthesis of the theoretical framework, the intervention approach, the evaluation system, and the intervention outcomes will follow the study selection.

## 2 Methods

A systematic review was conducted using the PRISMA protocol (Moher et al., 2009), and the literature review included four aspects of career interventions for high school students. (1) Theoretical foundations. (2) Intervention approaches. (3) Assessment. (4) Outcomes.

Five keywords and five eligibility criteria were defined to ensure the quality of the literature. The 5 keywords followed Whiston et al. (2017) and were combined by the search terms “career intervention” OR “career counselling” OR “career education” OR “career guidance” AND “high school students,” and both appeared in the title, abstract, or keywords. According to the literature, our study population was high school students, so we used “career” and did not use “occupational” or “vocational.” The five eligibility criteria included language, year, population, content, and assessment of the career intervention.

A comprehensive search was conducted on “Web of Science, Proquest, and EBSCO” in three databases. The search was limited to scientific papers written in English. Specifically, literature was searched from 2014 to 2024. Inclusion criteria included (1) the study population was high school students, (2) the study design was the career intervention, and (3) quantitative or qualitative data were provided to assess the effectiveness of the intervention. Exclusion criteria included (1) non-high school student population, (2) non-review articles, and (3) no focus on content of intervention.

First, two researchers analyzed the title and abstract of each article. The purpose was to find out if the study used high school students as the study population for the career intervention and intervention evaluation. The screened articles were further analyzed and discussed to obtain the final meta-analysis. Finally, summaries and discussions were made based on the theoretical framework, intervention approach, evaluation, and outcome.

## 3 Results

### 3.1 Main findings

**Figure 1:**
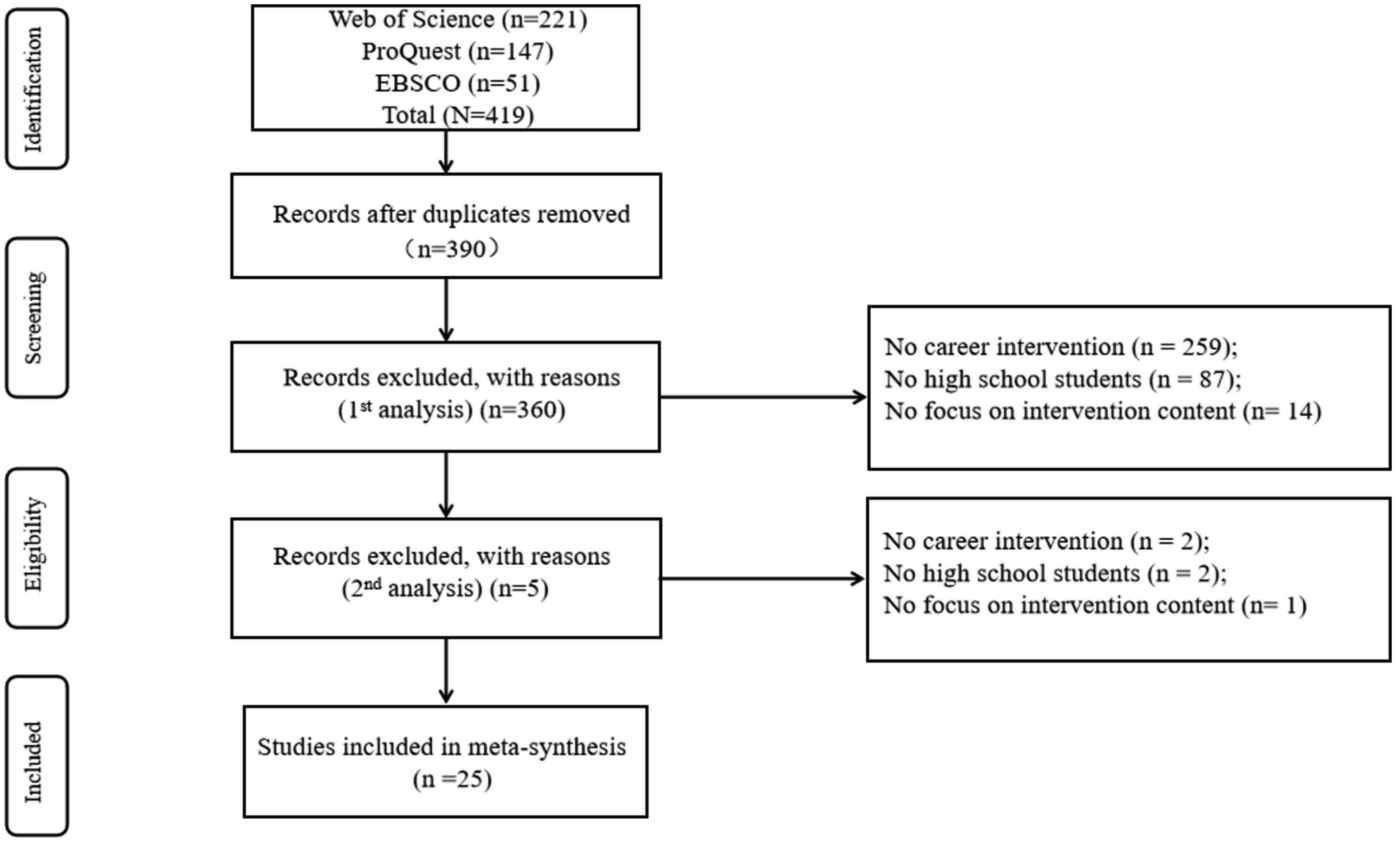
PRISMA flow chart.

419 articles were identified through the database search. These articles were first eliminated from duplication. Three hundred ninety (93%) non-duplicate articles were screened based on abstracts. The screening criteria for the first phase consisted of three components: whether or not it was a career intervention (*n* = 259, 66.4%), whether or not it was high school students (*n* = 87, 22.3%), and whether or not it focused on the content of the intervention (*n* = 14, 3.6%). The remaining 30 articles (7.6%) proceeded to the second screening stage, where 5 (16.7%) articles were excluded based on the same eligibility criteria (Figure 1), leaving 25 (83.3%) for meta-synthesis. Table 1 describes the theoretical underpinnings of the 25 studies, the structure of the interventions, the assessment system, and the results of the interventions. Of the included studies, the number of studies published on career interventions for high school students has grown recently, with the most significant number of career intervention studies in 2022 (*n* = 6).

**Table 1:** Research summary.

Theoretical	Intervention	Evaluation	Outcomes
(Janeiro et al., 2014) Do not specify	Group 1: Career counseling (6 sessions, 90 min each); Group 2: Career course (a single session, 90 min)	Pre- and post-test; 2 intervention groups	Career coping profiles; Career adaptability
(Maree and Symington, 2015) Career construction theory	Group career counseling: life design (8 sessions, 45–60 min each)	Students’ perceptions	Career adaptability
(Miles and Naidoo, 2016) Social Cognitive Career Theory	Group career counseling (5 sessions, 9 h)	Pre- and post-test; 8-week follow-up test; 1 intervention and 1 control groups	Career decision-making self-efficacy
(Cook and Maree, 2016) Career construction theory	Group 1: Career counseling (8 sessions, 45 min each); Group 2: Life Orientation (8 sessions, 45 min each)	Students’ perceptions	Career adaptability
(Dou et al., 2016) Cognitive Information Processing theory	Career education course (4 sessions)	Pre- and post-test; 1 intervention and 1 control groups	major decision-making self-efficacy and decision-making difficulty
(Chiesa et al., 2016) career decision-making self-efficacy theory	Group career counseling (6 sessions, 17 h)	Pre- and post-test	career decision-making self-efficacy; career exploration and career choice anxiety
(Atli, 2016) trait-factor theory	Career Guidance and Counseling Applications course (3 sessions)	Pre- and post-test	career maturity; career indecisiveness
(Maree et al., 2017) Career construction theory	Group 1: Career counseling (8 sessions, 45 min each); Group 2: Life Orientation (8 sessions, 45 min each)	Pre- and post-test; 1 intervention and 1 control groups	Career adaptability
(Maree, 2019) Career Construction theory	group career counseling (2 sessions: 8 h and 4.5 h)	Pre- and post-test	Career Adaptability; career-life themes and vocational interests and confidence
(Cardoso et al., 2018) Career construction theory	Group 1: My Career Story (7 sessions, 50 min each); Group 2: citizenship education (7 sessions, 50 min each)	Pre- and post-test; 1 intervention and 1 control groups	career certainty; career decision-making self-efficacy; career adaptability
(Gu et al., 2020) Do not specify	Career educational course (5 sessions, 45 min each)	Pre- and post-test; 1 intervention and 1 control groups	career decision self-efficacy; career decision-making difficulties
(Cadaret and Hartung, 2020) Career Construction theory	Group career construction counseling (3 sessions: 50–90 min each)	Pre- and post-test; 2 intervention groups	Career adaptability; Vocational identity
(Santilli et al., 2020) Career construction theory	group career guidance (3 sessions: 2 h each)	Pre- and post-test; 1 intervention and 1 control groups	career adaptability; future investment; wishes about the feature
(Chen, 2020) Career construction theory	Individual career counseling (4 sessions, 60 min each)	Students’ perceptions	life style
(Lau et al., 2021) vocational development theory	Group 1: career exploration intervention (8 sessions, 90 min each); Group 2: scheduled curriculum (8 sessions, 90 min each)	Pre- and post-test; 4-week follow-up test; 1 intervention and 1 control groups	Career maturity; self-concept
(Gülşen et al., 2021) Career construction theory	Group 1: career construction course (5 sessions: 75 min and 60 min each); Group 2:stress and test anxiety (50 min)	Pre- and post-test; 1 intervention and 1 control groups	future orientation; career adaptability; life satisfaction
(Cardoso et al., 2022) Career construction theory	Group 1:Life Design (8 sessions:50 min each); Group 2: regular classes (8 sessions:50 min each)	Pre- and post-test; 3-month follow-up test; 1 intervention and 1 control groups	Vocational identity; career adaptability; career decision-making self-efficacy
(Chen et al., 2022) Cognitive Information Processing theory	online career course (5 sessions: 45 min each)	Pre- and post-test; 1 intervention and 1 control groups	Career maturity; vocational identity; career decision-making difficulties
(Wang and Liu, 2022) Social Cognitive Career Theory; Cognitive Information Processing theory	Group 1: Career course (8 sessions, 45 min each); Group 2: Group counseling (8 sessions, 45 min each)	Pre- and post-test; 3-month follow-up test; 2 intervention and 1 control groups	Career adaptability
(Deng et al., 2020) Do not specify	Career education course (10 sessions, 40 min each)	Pre- and post-test; 6-month follow-up test; 1 intervention and 1 control groups	Career adaptability
(Garcia et al., 2021) Social Cognitive Career Theory	computer-based career course (2 sessions, 43 min and 90 min)	Pre- and post-test; 1 intervention and 1 control groups	vocational skills self-efficacy; outcome expectations; career decision-making difficulties
(Siebert et al., 2023) Do not specify	Group career counseling (6 sessions, 45 min each)	Pre- and post-test; 1 intervention and 1 control groups	proactive decision-making; career choice self-efficacy
(Jemini Gashi et al., 2023) social cognitive career theory	career guidance workshop (5 sessions, 2 days)	Pre- and post-test; 1 intervention group	Career self-efficacy; outcome expectations; career goals
(Maree and Magere, 2023) Career construction theory	Group career construction counseling (10 sessions, 880 min)	Students’ perceptions	Career decision-making difficulties; Career adaptability
(Wang and Liu, 2023) Career construction theory	Individual career counseling (3 sessions, 60 min each)	Students’ perceptions	Career adaptability; career decision-making self-efficacy

The country distribution of the study was as follows: China, 28% (*n* = 7); South Africa, 20% (*n* = 5); Portugal, 16% (*n* = 4); United States, 8% (*n* = 2); Malaysia, 4% (*n* = 1); Kosovo, 4% (*n* = 1); Germany, 4% (*n* = 1); Tanzania, 4% (*n* = 1); Turkey, 4% (*n* = 1); Italy, 4% (*n* = 1); Northern Cyprus, 4% (*n* = 1). 1; Northern Cyprus, 4% (*n* = 1). In terms of journals, most of the studies were published in The Career Development Quarterly (*n* = 3, 12%), Journal of Career Development (*n* = 3, 12%), International Journal of Adolescence and Youth (*n* = 3, 12%), Frontiers in Psychology (*n* = 3, 12%). Others appeared in the Chinese Journal of Clinical Psychology (*n* = 2, 8%), Journal of Vocational Behavior (*n* = 1, 4%), Journal of Psychology in Africa (*n* = 1, 4%), South African Journal of Education (*n* = 1, 4%), South African Journal of Psychology (*n* = 1, 4%), Journal of Counseling & Development (*n* = 1, 4%), Universal Journal of Educational Research (*n* = 1, 4%), British Journal of Guidance and Counselling (*n* = 1, 4%), Sustainability (*n* = 1, 4%), Cypriot Journal of Educational Sciences (*n* = 1, 4%), Journal of Innovative Education (*n* = 1, 4%) and Scientific Research in education (*n* = 1, 4%).

### 3.2 Theoretical framework

Of the 25 articles included, four (16%) do not detail the theoretical framework supporting the application of career intervention (Gu et al., 2020; Deng et al., 2020; Janeiro et al., 2014; Siebert et al., 2023). The remaining articles consisted of three main theories: the career construct theory mentioned in 12 (48%) articles (Cardoso et al., 2022; Maree and Magere, 2023; Maree, 2019; Cadaret and Hartung, 2020; Santilli et al., 2020; Chen, 2020; Maree and Symington, 2015; Cook and Maree, 2016; Maree et al., 2017; Cardoso et al., 2018; Gülşen et al., 2021; Wang and Liu, 2023). Social cognitive career theory was also mentioned in three (12%) articles (Jemini Gashi et al., 2023; Miles and Naidoo, 2016; Garcia et al., 2021). The cognitive information processing theory is mentioned in two (8%) articles (Dou et al., 2016; Chen et al., 2022). Moreover, one study compared the social cognitive career theory and the cognitive information processing theory of career interventions (Wang and Liu, 2022). In addition, other theories were also mentioned: vocational development theory (Lau et al., 2021), trait-factor theory (Atli, 2016) and career decision-making self-efficacy theory (Chiesa et al., 2016).

### 3.3 Intervention modality

Of the articles included, the intervention modalities consisted of five primary types: career group counseling, career course, individual group counseling, workshop and computer-based online intervention. Half of the studies mentioned the use of group career counseling (n = 12, 48%) (Janeiro et al., 2014; Miles and Naidoo, 2016; Cook and Maree, 2016; Chiesa et al., 2016; Maree et al., 2017; Maree, 2019; Santilli et al., 2020; Lau et al., 2021; Cardoso et al., 2022; Wang and Liu, 2022; Siebert et al., 2023; Maree and Magere, 2023), followed by workshops (*n* = 5, 20%) (Maree and Symington, 2015; Cardoso et al., 2018; Cadaret and Hartung, 2020; Gülşen et al., 2021; Jemini Gashi et al., 2023) and career courses (*n* = 4, 16%) (Dou et al., 2016; Atli, 2016; Gu et al., 2020; Deng et al., 2020). Less common were computer-assisted interventions (Garcia et al., 2021) (*n* = 1, 4%), online career courses (Chen et al., 2022) (*n* = 1, 4%), and individual career counseling (Chen, 2020; Wang and Liu, 2023) (*n* = 2, 8%).

Regarding the number and duration of sessions, most studies had 8 sessions (Maree and Symington, 2015; Cook and Maree, 2016; Maree et al., 2017; Lau et al., 2021; Cardoso et al., 2022; Wang and Liu, 2022). From 2 sessions (Garcia et al., 2021; Maree, 2019) to 10 sessions (Deng et al., 2020; Maree and Magere, 2023), each session lasted about 45 min. The maximum duration was 880 min (Maree and Magere, 2023). Only one article reported only the number of sessions and no duration (Atli, 2016).

Most of the high school students’ career intervention topics were self-knowledge, career knowledge, and decision-making. Overall, the sessions covered the following areas: Career world of information (Janeiro et al., 2014; Miles and Naidoo, 2016; Chiesa et al., 2016; Atli, 2016; Lau et al., 2021; Garcia et al., 2021; Jemini Gashi et al., 2023), exploration of personal interests (Miles and Naidoo, 2016; Chiesa et al., 2016; Atli, 2016; Gu et al., 2020; Lau et al., 2021; Chen et al., 2022; Garcia et al., 2021; Jemini Gashi et al., 2023), career planning (Janeiro et al., 2014; Lau et al., 2021; Chen et al., 2022), CV creation and interview training (Deng et al., 2020; Jemini Gashi et al., 2023), career goal exploration (Miles and Naidoo, 2016; Lau et al., 2021), self-efficacy and outcome expectations (Wang and Liu, 2022), career decision-making training (Janeiro et al., 2014; Miles and Naidoo, 2016; Dou et al., 2016; Gu et al., 2020; Chen et al., 2022). metacognition (Dou et al., 2016), reflecting on goals and choices (Siebert et al., 2023), and reflecting on personal career values (Chiesa et al., 2016), inclusively looking to the future (Santilli et al., 2020), to relate self-knowledge to educational opportunities (Cardoso et al., 2022).

### 3.4 Evaluation and intervention outcomes

Overall, studies included evaluation systems using pre- and post-tests (*n* = 20, 80%) (Dou et al., 2016; Chiesa et al., 2016; Atli, 2016; Maree et al., 2017; Maree, 2019; Cardoso et al., 2018; Gu et al., 2020), including an experimental group and a control group (*n* = 16, 64%) (Siebert et al., 2023; Garcia et al., 2021; Deng et al., 2020; Chen et al., 2022; Gülşen et al., 2021; Santilli et al., 2020). Four studies (16%) reported only one (Jemini Gashi et al., 2023) or two (Janeiro et al., 2014; Cadaret and Hartung, 2020; Wang and Liu, 2022) intervention groups. As for follow-up monitoring of intervention outcomes, only five (20%) studies had a tracking design (Miles and Naidoo, 2016; Deng et al., 2020; Wang and Liu, 2022; Lau et al., 2021; Cardoso et al., 2022). Follow-up results from two studies showed no maintenance of better outcomes (Miles and Naidoo, 2016; Deng et al., 2020). Under qualitative methods, the majority of the five (20%) studies used a life-design interview method focusing on assessing students’ perceptions that the study promotes the development of career adaptability in high school students (Maree and Symington, 2015; Cook and Maree, 2016; Chen, 2020; Maree and Magere, 2023; Wang and Liu, 2023). In addition, one (4%) study (Maree, 2019) utilized a mixed methods quantitative and qualitative intervention where qualitative (Career Interest Profile, Maree Career Matrix) and quantitative data (CAAS-SA) were collected simultaneously. In the following, the indicators and results of career interventions for high school students and strategies are described in detail.

### 3.5 Career adaptability

Career adaptability was the most evaluated indicator among the included studies and was categorized into career adaptability and career maturity. There are three types of measuring tools: (a) The Career Maturity Inventory- Form C (Janeiro et al., 2014; Cardoso et al., 2018); Career Maturity Scale (Atli, 2016); Career Maturity Inventory (Lau et al., 2021; Chen et al., 2022), (b) Career Adapt-Abilities Scal-Short Form (Gülşen et al., 2021), (c) Career Adapt-Abilities Scale-South African (Maree et al., 2017; Maree, 2019); Career Adapt-Abilities Scale-China Form (Wang and Liu, 2022; Deng et al., 2020); Career Adapt – Abilities Scale the Portuguese version (Cardoso et al., 2022).

Overall, the intervention group showed higher career adaptability (Santilli et al., 2020; Cadaret and Hartung, 2020; Gülşen et al., 2021; Cardoso et al., 2022; Wang and Liu, 2022; Deng et al., 2020), career maturity (Janeiro et al., 2014). Two studies found no change in career adaptability after the career intervention. Research suggests that life design group counseling did not increase career adaptability in high school students compared to traditional career courses (Maree et al., 2017). Some researchers have also used an alternative life design approach, “My Career Story.” The results indicated that the life design intervention did not impact high school students’ career adaptability (Cardoso et al., 2018). For the intervention modality, Janeiro et al. (2014) compared the effects of a career course and a six-week group counseling on students with different career coping styles. The results showed that group counseling was more effective than the career course, which only increased career curiosity and self-confidence in one group of students. In contrast, group career counseling was able to have an impact on students with insecure career coping.

For qualitative methods, the research tools were used Life Design (Maree and Symington, 2015), Career Interest Profile (Cook and Maree, 2016; Maree, 2019; Maree and Magere, 2023), Maree Career Matrix (Maree, 2019), the Innovative Moments Coding System and Future Career Autobiography (Wang and Liu, 2023). The study found that qualitative methods can enhance the level of career adaptability of high school students.

### 3.6 Career decision-making

Career decision-making was the second most evaluated indicator in the included studies. It was categorized as follows: (a) career decision-making self-efficacy, (b) career decision-making difficulties, (c) career indecisiveness, (d) career certainty, and (e) career choice anxiety.

Category (a) used the Career Decision-making Self-Efficacy Scale-Short Form (Miles and Naidoo, 2016; Chiesa et al., 2016; Cardoso et al., 2022), the Career Decision Self-Efficacy Scale- Short Form (Cardoso et al., 2018), the Career Choice Resilience (Siebert et al., 2023), The Lack of Self-Knowledge subscale (Chiesa et al., 2016), and the Major Decision-Making Self-Efficacy Scale (Dou et al., 2016; Gu et al., 2020).

Category (b) used the Career Decision-Making Difficulties Questionnaire (Gu et al., 2020; Chen et al., 2022; Garcia et al., 2021) and the Major Decision-Making Difficulties Questionnaire (Dou et al., 2016). For their study, Dou et al. (2016) developed items to assess high school students’ major decision-making abilities. The Major Decision-Making Self-Efficacy Scale and the Major Decision-Making Difficulty Scale were administered to assess categories (a) and (b) (Dou et al., 2016). Category (c) has been assessed career indecisiveness by the Career Decision Scale (Atli, 2016). Category (d) has been assessed by the Vocational Certainty Scale (Cardoso et al., 2018). Finally, career choice anxiety has been assessed for category (e) by the Career Choice Resilience (Chiesa et al., 2016).

Overall, the intervention group demonstrated higher career decision-making self-efficacy (Miles and Naidoo, 2016; Chiesa et al., 2016; Cardoso et al., 2022; Cardoso et al., 2018), major decision-making self-efficacy (Dou et al., 2016), certainty (Cardoso et al., 2018) as well as career decision-making difficulty (Gu et al., 2020; Chen et al., 2022; Garcia et al., 2021), major decision-making difficulty (Dou et al., 2016), and career indecisiveness (Atli, 2016). Two studies found no changes after career intervention for career choice anxiety (Chiesa et al., 2016) and major decision-making self-efficacy (Gu et al., 2020).

### 3.7 Vocational identity

Vocational identity was assessed through The Vocational Identity Scale (Cardoso et al., 2022), the Vocational Identity Measure (Cadaret and Hartung, 2020), and the Vocational Identity Status Assessment (Chen et al., 2022). Of these, the Vocational Identity Status includes two subscales, the Career Exploration and the Career Commitment, which refer to students’ commitment to the process of transitioning to a worker role. Overall, these studies suggest that career interventions promote the development of vocational identity among high school students.

### 3.8 Outcome expectations

Outcome expectancy, a concept from Social Cognitive Career Theory that implies what will happen in career-related areas, was assessed in three studies. Notably, Chiesa et al. (2016) reviewed the literature and found no measurement tools for outcome expectations. The follow-up researchers used the Vocational Outcome Expectations Revised (VOE-R) Scale (Garcia et al., 2021). Another study assessed learners’ assessment of career-related behaviors and expectations of outcomes (Jemini Gashi et al., 2023). Both studies showed an increase in outcome expectations for participants after the intervention.

### 3.9 Other evaluations

Janeiro et al. (2014) measured students’ career coping styles using the Time Perspective Inventory (TPI), the Career Attributions Beliefs Scale (CABS), and the Coopersmith Self-Esteem Inventory. The results showed that a six-week career intervention was more effective for students with insecure, pessimistic, or superficial career coping styles. Career Exploration uses the Career Exploration subscale of the Career Development Inventory as a measurement tool to assess whether and where to search for information for career development. The study was conducted to improve students’ career exploration behaviors by increasing their career decision-making self-efficacy. Hierarchical linear modeling indicated that the intervention had a significant effect on improving career exploration (Chiesa et al., 2016). Lau et al. (2021) used the Tennessee Self-Concept Scale to measure six dimensions of self-concept, including physical, moral, personal, family, social, and academic/work. Self-concept improved immediately after the intervention, and the effects were maintained after 4 weeks. Gülşen et al. (2021) assessed future orientation and resilience using the “Designing My Future” scale. Future orientation refers to an individual’s hopes, thoughts, plans, motivations and feelings about the future. Resilience refers to the ability to cope with challenging life experiences. Significant differences were observed in terms of gender. High school girls’ scores were higher than those of boys for future orientation and resilience. Life satisfaction is defined as the cognitive component of subjective well-being. Gülşen et al. (2021) suggest that career interventions have a negligible effect on life satisfaction. It may be caused by the fact that personality traits and life events can influence subjective well-being.

## 4 Discussion

This study systematically reviewed the content of career interventions for high school students. Twenty-five articles met the inclusion criteria, followed by a detailed description of the theoretical framework, intervention approach, evaluation system, and outcomes generated.

### 4.1 Theoretical framework

The theoretical framework of career intervention for high school students consists of Career Construction Theory, Social Cognitive Career Theory, and Cognitive Information Processing Theory. Overall, the forms of intervention for career construction theory and social cognitive career theory are group counseling, while cognitive information processing theory interventions are career courses. Wang and Liu (2022) compared the two theories of career intervention. They showed that the social cognitive career theory had a more significant impact on high school students than the cognitive information processing theory. The focus of career development for high school students is to develop career adaptability to better adapt to changes in the outside world. The researcher concluded that career counseling based on social cognitive career theory can increase the career adaptability of high school students. This reveals that future high school career education can be developed through the social cognitive career theory framework in the borderless career era.

The career construct theory is the most used theory for high school career interventions. The theory consists of three components: vocational personality, career adaptability, and life theme. Career construction reflects the interaction between the individual and the environment, in which the individual subjectively adapts to the changing environment. The process of career construction is a continuous process of developing and implementing self-concept. The development of career constructs can be facilitated through dialog that explains career development tasks and career transitions, exercises to enhance adaptability, and ways to validate career self-concepts (Savickas, 2005). The main components of career construct theory on high school career group interventions are providing information, enriching extracurricular activities, and alternative experiences. A common approach to identifying career themes for individual counseling includes five questions: role models, interest activities, favorite stories, mottos and early memories (Gao and Qiao, 2022).

### 4.2 Intervention modality

With regard to intervention methods, group counseling is dominant. Individual and group counseling are effective methods, but at the same time, it is essential to consider the numbers and the financial benefits that interventions can bring (Whiston, 2011). Having the support of others in career decision-making is a crucial factor, and group counseling enables access to provide a supportive environment for individual growth (Whiston et al., 2017). More importantly, groups are cost and time-effective (Langher et al., 2018). In general, most high school group counseling sessions last around eight sessions of 45 min each. Ryan (1999) suggest that career counseling should last four to five sessions. Whiston et al.'s (2017) meta-analysis came to a similar conclusion. High school students require more extended sessions, possibly due to their lack of career knowledge and experience.

Additionally, the thematic content of high school group counseling was consistent with previously obtained critical components of the meta-analysis. Ryan (1999) five key components of career counseling were operating manuals and writing exercises, personal feedback, information about the professional world, role modeling, and providing support. Whiston et al.'s (2017) meta-analysis of the three key components were counselor support, values clarification and psycho-educational interventions.

Career intervention modes mainly include career courses, group counseling, and individual counseling for high school students. Each intervention mode has its advantages. Career courses can meet the career development needs of all students. The curriculum is more systematic, extensive and comprehensive. Moreover, there is no strict requirement on the number of participants, which is easy to promote and operate. Group and individual career counseling, though limited in terms of the number of participants, are more targeted and can meet the career development needs of individual students.

Therefore, it is possible to design a career development model for senior secondary students. The first tier is open to all students, and the career curriculum is designed according to students’ needs. The first tier targets students with career development needs with career group counseling. Tier 3 focuses on students with special developmental needs and develops individual career counseling.

### 4.3 Evaluation

Regarding the evaluation of interventions, studies have mainly used quantitative with pre- and post-tests in experimental and control groups. Qualitative studies have primarily used interviews to assess changes in students’ perceptions. Maree et al. (2017) used a mixed-methods intervention research design to explore the value of group counseling using CIP and MCM, and it investigated whether there was a strong positive correlation between qualitative and quantitative data outcomes. Overall, the results of qualitative group career counseling were more positive than the quantitative. Therefore, future research could use a mixed design with qualitative assessments to complement quantitative assessments. Notably, there are fewer follow-up studies of career interventions. However, the intervention failed to achieve a better maintenance effect (Miles and Naidoo, 2016; Deng et al., 2020). Therefore, future studies should focus on the sustained effects of the intervention.

### 4.4 Outcome of the intervention

The results regarding the intervention also confirmed previous meta-analysis studies (Whiston et al., 2017). Expressly, the results of the present study indicated that there were positive effects in the areas of career adaptability, career decision-making, career identity, career exploration, self-efficacy, and expectations for the future. In these studies, the outcome of the intervention has focused on career adaptability and career decision-making.

Recent systematic reviews (Vashisht et al., 2021) and meta-analyses (Stead et al., 2022) have similarly demonstrated a concern for assessing career adaptability and career decision-making skills. The results seem to indicate that there is little change in the outcomes of career interventions over time. Therefore, it is essential for high school students in the era of borderless careers to develop career adaptability to cope with changing environments, and it is also necessary to increase high school students’ career decision-making self-efficacy and career certainty (Cardoso et al., 2018).

The dramatic increase in the use of career decision self-efficacy as an outcome measure has been attributed to the growing popularity of Social Cognitive Career Theory and the development of measurement tools in the field (Stead et al., 2022). However, Chiesa et al.'s (2016) literature review did not identify measurement tools that measured outcome expectations. Measurement tools may limit the development of social cognitive career interventions. Therefore, there is a need to develop measurement tools for social cognitive career theory in the future.

Another issue that stood out was the importance of conducting research to assess interventions for special populations of high school students. In general, research has not considered career support for students in foster care, transfer, and learning disabilities to help them address career issues in their development and reduce career barriers for this population.

## Data Availability

The original contributions presented in the study are included in the article/supplementary material, further inquiries can be directed to the corresponding author.
